# Quantitative detection of human Malawi polyomavirus in nasopharyngeal aspirates, sera, and feces in Beijing, China, using real-time TaqMan-based PCR

**DOI:** 10.1186/s12985-017-0817-2

**Published:** 2017-08-14

**Authors:** Fen-lian Ma, Dan-di Li, Tian-li Wei, Jin-song Li, Li-shu Zheng

**Affiliations:** 1 Key Laboratory for Medical Virology, Ministry of Health, National Institute for Viral Disease Control and Prevention, China CDC, 100 Ying-Xin St., Xi-Cheng District, Beijing, 100052 China; 2 Department of Pediatrics, Beijing Friendship Hospital, Capital Medical University, Beijing, 100052 China

**Keywords:** Human Malawi polyomavirus (MWPyV), TaqMan real-time PCR, Nasopharyngeal aspirate, Feces, Respiratory virus

## Abstract

**Background:**

Human Malawi polyomavirus (MWPyV) was discovered in 2012, but its prevalence and clinical characteristics are largely unknown.

**Methods:**

We used real-time TaqMan-based PCR to detect MWPyV in the feces (*n* = 174) of children with diarrhea, nasopharyngeal aspirates (*n* = 887) from children with respiratory infections, and sera (*n* = 200) from healthy adults, and analyzed its clinical characteristics statistically. All the MWPyV-positive specimens were also screened for other common respiratory viruses.

**Results:**

Sixteen specimens were positive for MWPyV, including 13 (1.47%) respiratory samples and three (1.7%) fecal samples. The samples were all co-infected with other respiratory viruses, most commonly with influenza viruses (69.2%) and human coronaviruses (30.7%). The MWPyV-positive children were diagnosed with bronchopneumonia or viral diarrhea. They ranged in age from 12 days to 9 years, and the most frequent symptoms were cough and fever.

**Conclusions:**

Real-time PCR is an effective tool for the detection of MWPyV in different types of samples. MWPyV infection mainly occurs in young children, and fecal–oral transmission is a possible route of its transmission.

**Electronic supplementary material:**

The online version of this article (doi:10.1186/s12985-017-0817-2) contains supplementary material, which is available to authorized users.

## Background

The family *Polyomaviridae* contains small encapsidated DNA viruses with a closed circular DNA genome of ~5 kb, packaged within an icosahedral capsid structure. The regulatory proteins, large T and small T antigens, are encoded in the early region of the genome, and structural proteins VP1, VP2, and VP3 are encoded in the late region [1]. Human polyomaviruses (HPyVs) usually cause asymptomatic primary infections, and persist in the body throughout life [2]. Thirteen HPyVs have been identified and successively isolated from urine (BKPyV), brain tissue (JCPyV), respiratory secretions (KIPyV and WUPyV), skin (MCPyV, HPyV6, HPyV7, and TSPyV), serum (HPyV9), stool samples (HPyV10 and isolates MW and MX, STLPyV), liver tissue (HPyV12), and vascular endothelial cells (NJPyV). The detection of HPyVs in these tissues suggests there exists an unrecognized tropism for HPyV [3]. HPyV10 is the type species of the genus *Deltapolyomavirus* in the family *Polyomaviridae*. In 2012, human polyomavirus 10 (HPyV10), MW polyomavirus (MWPyV), and MX polyomavirus (MXPyV) were isolated from condylomas on the buttocks of patients, fecal specimens from healthy children, and stool samples from children suffering diarrhea, respectively [4–6]. The whole-genome sequences of MXPyV, MWPyV, and HPyV10 are nearly identical, indicating that they are variants of the same species, which suggests that HPyV10 is a fecal contaminant rather than a wart-causing skin virus [7]. HPyV10 has not yet been definitively linked to a particular human disease [8]. Very little is known about MWPyV, so it is important to investigate its prevalence, basic epidemiology, genomic variability, and possible pathogenicity.

Unlike conventional PCR-based approaches, real-time quantitative PCR (qPCR) uses a fully automated detection system, with high sensitivity. Different chemistries are commercially available for qPCR, including SYBR Green, TaqMan, Scorpion, and Molecular Beacons, but TaqMan is most frequently used because it can discriminate sequences that differ by only one nucleotide [9]. In a previous study, we demonstrated the specificity, sensitivity, and reproducibility of a TaqMan-based real-time qPCR for detecting HPyV6 [10]. Therefore, in this study, we used real-time qPCR and DNA sequencing to investigate the presence of MWPyV in fecal samples from 174 children with diarrhea, nasopharyngeal aspirate (NPA) samples from 887 children with acute respiratory tract infections (ARIs), and sera from 200 healthy adults in China.

## Materials and methods

### Study population and samples

NPA samples were collected from 887 hospitalized children with ARIs, who ranged in age from 3 days to 14 years, at Beijing Friendship Hospital, China, between October 2011 and September 2012. A total of 174 fecal samples were collected from children <5 years old presenting with viral diarrhea at the Capital Institute of Pediatrics, China, in 2015. The sera of 200 healthy adults, from the Institute of Blood Transfusion, Chinese Academy Medical Sciences, were collected between October 2011 and June 2011. All specimens were collected and transported immediately to the National Institute for Viral Disease Control and Prevention, China CDC, and stored at −80 °C until processing.

### Nucleic acid extraction

Fecal samples were diluted approximately 1:6 in phosphate-buffered saline and filtered through membranes with a pore size of 0.45 μm before the DNA was extracted. The total nucleic acids were extracted from 200 μl of the fecal, serum, and NPA samples with a QIAamp MinElute Virus Spin Kit (Qiagen, Beijing, China; catalogue no. 57704) and eluted in a volume of 100 μl, according to the manufacturer’s instructions. The DNA concentrations were determined with a BioPhotometer™ plus (Eppendorf, Germany) at a wavelength of 260 nm (A_260_), and DNA purity was evaluated with the A_260_/A_280_ ratio. The samples were stored at −80 °C for subsequent use.

### Construction of a standard curve

A TaqMan real-time qPCR assay was designed to target the MWPyV VP1 gene using the Real time PCR tool (Integrated DNA Technologies, Inc.). The binding sites for the primers and probes used for MWPyV share 100% sequence identity with the VP1 sequences reported for HPyV10 and MXPyV. The primers and probe used for this assay were 5′-GGAGACCCCACTTTAACTAGAC-3′ (forward primer), 5′-ACTTGGCATATCTGTTACTGGG-3′ (reverse primer), and 5′-FAM-ATATGGATTTGCGTTGCTGCCCG-TAMRA-3′ (TaqMan probe, where FAM is TAMRA). The resulting amplicon was 72 bp in length. The qPCR was performed in a total volume of 20 μl containing 10 μl of 2 × TaqMan Gene Expression Master Mix (Takara, Japan), 1.8 μl of each primer (10 pmol/μl), 0.2 μl of probe (10 pmol/μl), 4.2 μl of diethyl-pyrocarbonate-treated water, and 2 μl of pMD18-T/VP1 plasmid (plasmid pMD18-T linked to the 72-bp target sequence). The samples were tested in a 96-well-plate format, with sterile water as the negative control. The cycling conditions were 50 °C for 2 min, 95 °C for 10 min, followed by 40 cycles of 95 °C for 15 s and 60 °C for 1 min, using the Mx3005P qPCR System (Agilent Stratagene, USA). Serial 10-fold dilutions of pMD18-VP1 were prepared, with the standard plasmid serially diluted from 10^10^ to 10^0^ copies/μl. The dilutions were added to the real-time qPCR reactions, and analyzed in triplicate. The results were used to construct a standard curve. The specificity of the qPCR was assessed by also testing WUPyV, KIPyV, JCPyV, BKPyV, and HPyV7. Reproducibility was evaluated by adding 10^5^–10^8^ copies/μl pMD18-T/VP1 to each reaction, and performing all the reactions four times, with the housekeeping gene glyceraldehyd-3-phosphate dehydrogenase as the internal control.

### Virus detection

The nucleic acid (2 μl) from each NPA, fecal, or serum sample was added to a real-time qPCR reaction. The Ct values were measured as the point at which the fluorescent signal of the sample crossed the threshold value. All the MWPyV-positive NPA specimens were also screened for *Influenza A virus* and *Influenza B virus*, parainfluenza viruses (PIVs) 1–4, human metapneumovirus (HMPV), respiratory syncytial virus (RSV) and human coronaviruses (HCos) NL63, OC43, 229E, and HKU1, using the AgPath-ID™ One-Step RT–PCR Reagent (Invitrogen, Life Technologies Inc.), and for human bocavirus (HBoV) using TaqMan real-time qPCR. The AgPath-ID™ One-Step RT–PCR cycling program was: 55 °C for 10 min, 95 °C for 10 min, followed by 40 cycles of 95 °C for 15 s and 60 °C for 45 s. A Ct value of ≤40 was confirmed with DNA sequencing.

### DNA sequencing

All qPCR-positive samples were tested with conventional PCR using the primers described. The amplified PCR products were cloned into the pMD18-T vector (Takara) for sequencing (Invitrogen, Life Technologies Inc.). The complete VP1 gene of MWPyV was amplified with nested PCR in a 25 μl reaction volume that contained 2.5 μl of 10 × Ex Taq buffer (Takara), 1 μl of forward primer (10 pmol/μl), 1 μl of reverse primer (10 pmol/μl), 2 μl of dNTP mix (2.5 mmol/L), 0.5 μl of Ex Taq DNA Polymerase (Takara), 2 μl of positive-sample nucleic acid, and 16 μl of sterile water. The primers used in this study are listed in Additional file 1: Table S2. The amplified products were cloned into pMD18-T for sequencing. The full-length VP1 sequence was aligned with the sequences of HPyVs available in GenBank with the nucleotide BLAST program. The sequences have been submitted to the GenBank database.

### Clinical data analysis

Clinical data, including the demographic data (age and sex), and the vital symptoms and signs at admission, were considered. Statistical comparisons were made with Fisher’s two-tailed exact test and the Mann–Whitney *U* test. All analyses were performed with SPSS 19.0 software.

### Nucleotide sequence accession numbers

The sequences determined in this study were submitted to GenBank under accession numbers KY774318, KY774319, KY774320 and KY774321.

## Results

### Standard curve construction for qPCR

To determine the analytical sensitivity, different concentrations of the plasmid pMD18-VP1, ranging from 10^10^ to 10^0^ copies/μl, were used. The slope of the standard curve was −3.28 and R^2^ = 0.994, the efficiency was 101.7 and the limit of detection was 10 copies. These parameters are suitable for quantifying the MWPyV load. The assay did not amplify any product from the other viral pathogens tested (WUPyV, KIPyV, JCPyV, BKPyV, and HPyV7), indicating that the primers and probe are specific for the detection of MWPyV. Four different DNA concentrations (10^5^–10^8^ copies per reaction) were each tested four times in each run. The maximum coefficient of variation was 0.75%, indicating good precision (data not shown).

### Epidemiology of MWPyV

MWPyV was only detected in respiratory and fecal specimens. With real-time qPCR, 13 (1.47%) of the 887 NPA specimens were positive for MWPyV; three (1.72%) of the 174 fecal samples were positive for MWPyV; and no MWPyV was detected in the serum specimens. The screening results for the various sample types are presented in Table 1.

**Table 1:** Viral loads and clinical characteristics of MWPyV-positive children

Sample	Number	Age (Months)	Sex	Date	Symptoms	Diagnosis	Copy Number	Ct Value
NPA	BJ20	60	M	Oct-2011	Fever, cough	Bronchopneumonia	549.54	34.31
NPA	BJ130	84	M	Dec-2011	Fever, cough vomiting	Bronchopneumonia	26.92	38.61
NPA	BJ242	11	M	Feb-2012	Fever convulsion	Bronchopneumonia rotavirus enteritis	4168.69	31.42
NPA	BJ289	29	F	Feb-2012	Fever, cough	Bronchopneumonia	371.54	34.87
NPA	BJ325	108	M	Mar-2012	Fever, cough vomiting	Bronchopneumonia	239.88	35.48
NPA	BJ531	48	F	May-2012	Fever, cough	Bronchopneumonia	112.02	36.59
NPA	BJ538	72	M	May-2012	Fever,tonsil hypertrophy	Bronchopneumonia Infectious mononucleosis	181.97	35.88
NPA	BJ541	12	F	May-2012	Fever, cough	Bronchopneumonia	60.26	37.45
NPA	BJ643	0.4	M	Jun-2012	Fever	Newborn pneumonia, newborn omphalitis, newborn pathological jaundice	37.15	38.14
NPA	BJ648	12	M	Jun-2012	Cough gasping	Bronchopneumonia Infant asthma	40.74	38.02
NPA	BJ813	24	F	Aug-2012	Fever, cough	Bronchopneumonia	48.98	37.75
NPA	BJ817	24	M	Aug-2012	Fever, cough	Influenza	58.88	37.51
NPA	BJ886	108	M	Sep-2012	Cough	Bronchopneumonia	63.1	37.4
Feces	BJ193	11	M	Jan-2015	Fever, diarrhea	Viral diarrhea	87.1	36.93
Feces	BJ269	4	F	Jun-2015	Fever, diarrhea	Viral diarrhea	46.77	37.84
Feces	BJ1101	20	M	Feb-2015	Fever, diarrhea	Viral diarrhea	50.12	37.73

**Fig. 1:**
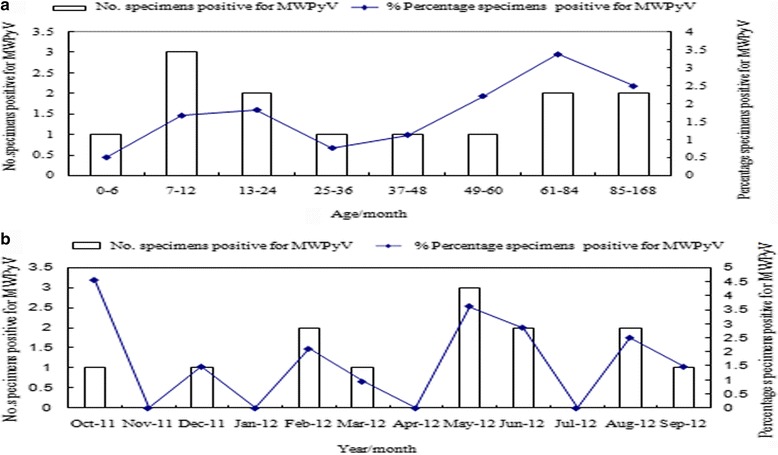
**a** Age distribution and the percentage of MWPyV-positive children with ARIs; **b** Seasonal distribution and the percentage of MWPyV-positive children with ARIs

Of the 887 children with ARIs included in the study, 524 were male, so the male:female ratio was 1.44:1. The age range was 3 days to 14 years. The MWPyV prevalence rate was 1.47% (13/887), and was higher in males (9/524, 1.7%) than in females (4/363, 1.0%). The infected children ranged in age from 12 days to 9 years, and children aged ≤5 years accounted for 69.23% (9/13) of the total MWPyV-positive children. Children aged 7–24 months had the highest infection rate (5/13, 38.5%), and the infection rate of those aged 61–168 months ranked second (4/13, 30.8%) (Fig. 1a). MWPyV was detected throughout the year, except in January, April, July, and November. The MWPyV infections were mainly sporadic: one case in October 2011, one case in December 2011, two cases in February 2012, one case in March 2012, three cases in May 2012, two cases in June 2012, two cases in August 2012, and one case in September 2012 (Fig. 1b). The peak incidence (1/22, 5.54%) occurred in October 2011.

The majority of fecal samples were collected in 2015, with approximately equal numbers of samples collected each month in the first half of the year, except that 1–3 samples were collected per month from July to October. Of the 174 children with diarrhea included in the study, 107 were males, so the male:female ratio was 1.6:1. The age range was 15 days to 5.75 years, and children aged 0.5–12 months accounted for 63.8% (111/174), and those aged 13–24 months accounted for 23.6% (41/174). The prevalence of MWPyV was 1.72% (3/174). Of the three positive patients, more were male (2/107, 1.87%) than female (1/67, 1.49%). The two infected male children were 11 and 20 months old, and the female was 4 months old, so all three MWPyV-positive patients were <2 years. These MWPyV infections occurred in January, February, and June.

Of the 13 MWPyV-positive NPA samples, 12 (92%) were co-infected, eight with one other respiratory virus and four with two other respiratory viruses. Influenza virus was detected most frequently (9/13, 69.2%), including *Influenza A virus* (61.5%) and *Influenza B virus* (7.7%), followed by human coronaviruses (HCo, 30.7%: HCo-OC43, 23%; HCo-NL63, 7.7%), human parainfluenza virus type 3 (PIV3, 15.4%), and human bocavirus (HBoV, 7.7%). One of the 13 samples (sample BJ242, 7.7%) was co-infected with *Rotavirus*. All the fecal specimens had already tested positive for rotavirus, and 2/3 (fecal samples BJ269 and BJ1101, 66.7%) were also co-infected with *Influenza A virus* (Table 2). No evidence indicated that the illnesses of the co-infected patients were more serious than those of patients with single MWPyV infections (results not shown).

**Table 2:** Co-detection of MWPyV and other common respiratory viruses

Sample	HBoV	HMPV	RSV	IFVA	IFVB	PIV1	PIV2	PIV3	PIV4	HCoV-229E	HCoV-OC43	HCoV-HKU1	HCoV-NL63
BJ20^#^				+									
BJ130^#^													+
BJ242^#^													
BJ289^#^				+							+		
BJ325^#^					+								
BJ531^#^				+									
BJ538^#^				+							+		
BJ541^#^								+					
BJ643^#^				+									
BJ648^#^				+				+					
BJ813^#^	+												
BJ817^#^				+							+		
BJ886^#^				+									
BJ193*													
BJ269*				+									
BJ1101*				+									

^#^represents NPA specimens; * represents fecal specimens; ^+^ represents positive; HBoV human bocavirus; HMPV human metapneumovirus; RSV respiratory syncytial virus; IFVA influenza virus A; IFVB influenza virus B; PIV1–4 parainfluenza virus types 1–4; HCoV 229E, OC43, HKU1, NL63, human coronaviruses 229E, OC43, HKU1, NL63

### Viral load and clinical characteristics of MWPyV in children

MWPyV-positive children with ARIs were diagnosed with bronchopneumonia (11/13, 84.6%), newborn pneumonia, newborn omphalitis, newborn pathological jaundice, infant asthma, influenza, rotavirus enteritis, or infectious mononucleosis (*n* = 1, 7.7%). The clinical symptoms of MWPyV-positive children with ARIs included fever (*n* = 12, 92.3%), which was the commonest symptom, cough (*n* = 10, 76.9%), vomiting (*n* = 2, 15.4%), and convulsion, tonsil hypertrophy, and gasping (*n* = 1, 7.7%). Using the standard curve method, we calculated the viral genome copy numbers in the positive NPA specimens to be 26.92–4168.69 copies/μl based on the previously established real-time PCR assay (Table 1). The genome copy number was 4168.69 copies/μl in one child with both bronchopneumonia and rotaviral enteritis, which was much higher than the 26.92 copies/μl NPA in children suffering bronchopneumonia and other diseases (*P* < 0.05, Mann–Whitney U test). The clinical symptoms of all three MWPyV-positive children with diarrhea included diarrhea and fever (100%). The viral genome copy numbers in the three positive fecal specimens, calculated with the standard curve, were 46.77, 50.12, and 87.10 copies/μl.

### DNA sequencing

The sequences of the 72-bp PCR products from all the MWPyV-positive NPA and fecal samples were 100% identical to the corresponding MWPyV, MXPyV, and HPyV10 sequences, according to a BLASTN comparison. To determine the complete genomic sequence of the VP1 gene, four genomic fragments were amplified with nested PCR using specific primers. The complete VP1 genomic sequences of only four MWPyV-positive NPA samples (BJ289, BJ531, BJ538, and BJ541) were amplified. DNA sequencing confirmed that these four positive samples contained MWPyV. The BJ289 NPA sample contained an MWPyV isolate that shared 100% sequence identity with strain MA095. The MWPyV isolates in samples BJ531, BJ538, and BJ541 shared 99% sequence similarity (5–10 single-base mutations) with isolates HN037, HB087C, HB040C, and HB039C.

## Discussion

Several studies have reported the widespread distribution of MWPyV in different areas of the world, including Africa, the USA, Asia, Italy, Australia, and South America [5, 11–15]. In this study, we used a qPCR assay to rapidly and accurately screen for MWPyV in NPA, serum, and fecal samples collected from different populations in China. Thirteen (1.47%) of the samples from children with ARIs and three (1.72%) from children with diarrhea were positive for MWPyV, whereas no serum (*n* = 200) was positive for MWPyV. In brief, the analysis of 1261 clinical samples only detected MWPyV in respiratory and fecal specimens from children, suggesting that the establishment of the primary infection occurs at an early age, and that the gastrointestinal and respiratory tracts are sites of viral persistence. This indicates a possible fecal–oral route of transmission during early childhood.

To verify the specificity of the primers and probe used in this study, other viruses (WUPyV, KIPyV, JCPyV, BKPyV, and HPyV7) were also tested, but no fluorescent signal was detected. We excluded any false-positive results by sequencing all the PCR products. It is noteworthy that all the 72-bp PCR products from positive samples shared 100% identity with the corresponding genomic regions of MWPyV, HPyV10, and MXPyV, accessed from GenBank. The sequences of the complete VP1 genes confirmed that NPA samples BJ289, BJ531, BJ538, and BJ541 contained MWPyV, and revealed that the viruses in the last three samples contained 5–10 single-base mutations. In total, 16 MWPyV-positive samples were detected in this study, and the cycle threshold (Ct) values for most of these were high (average Ct = 36.62, range 31.42–38.61), suggesting that the viral load was low. It is possible that the PCR failed to amplify the complete VP1 gene sequences from the other 12 positive samples.

Most ARIs are caused by viruses [16]. In this study, the prevalence of MWPyV in NPA was 1.47%. This is similar to findings in Australia (1.5%) [17], but differs from a study in Mexico, which reported a detection rate of 0.74% in nasal washes from children suffering ARI [6]. In a study by Rockett et al., MWPyV was the most prevalent polyomavirus (56%) detected in respiratory specimens from children with ARI. These findings are supported by other studies that frequently detected MWPyV in respiratory samples from acutely ill patients and babies with upper respiratory symptoms [13, 17]. Possible explanations for these discrepancies may be the use of different detection assays, differently defined cut-off values, different geographic regions examined, and the varying characteristics of the study populations.

MXPyV, MWPyV, and HPyV10 are presumably human tropic [5], and appear to be largely confined to the gastrointestinal tract [6]. For this reason, we tested fecal specimens from children with a diagnosis of viral diarrhea. We detected MWPyV in 1.72% of fecal specimens from these children, which is lower than previously reported rates of 2.3% and 5.9% in children with diarrhea in St Louis, USA, and Australia [5, 17]. These discrepancies are probably attributable to the different periods at which the samples were collected and the different detection methods used. The detection of MWPyV in the feces of children with diarrhea indicated that MWPyV may not be a strong candidate for gastroenteritis in children because of the co-infection with rotavirus.. WUPyV, KIPyV, SV40, BKV, JCV, MXPyV, and MCV have also been detected in human feces [6, 18–21], although their primary sites of pathology are elsewhere in the human body.

Seroepidemiology plays an important role in establishing the link between HPyVs and disease and in understanding the dynamics of infection. In this study, MWPyV was undetectable in the blood of healthy adults, consistent with other published data, and MWPyV was not previously detected in the blood of autoimmune children [15], as has also been observed for the MXPyV, JCPyV, and BKPyV [6]. However, in contrast to our findings, the seroprevalence of MWPyV in adulthood was 42% in a large sample from Italy [22], 66% in a sample from Colorado [14], 84% in 1614 serum samples from the Czech Republic [23], and almost 100% in samples from New Hampshire, USA [24]. These contrasting data may be attributable to the relatively small number of blood samples we analyzed (*n* = 200) or a different MWPyV may have been isolated from the human samples, to which have affected the antibody binding during serological analysis. Moreover, MWPyV DNA is more likely to be detected in blood during the primary infection than during viral reactivation [25]. In a study by Nicol et al., MWPyV was cleared or the virus was not reactivated with age [22], as has been reported for TSPyV [26], which may also explain why we detected no MWPyV-positive sera.

Factors such as age, sex, the time of year when multiple viruses circulate, and a history of immunosuppression are associated with an increased chance of viral co-infection [27]. In this study, 14 of the 16 MWPyV-positive patients were co-infected with other viruses, and four of these had multiple infections. The primers and probes used in this study are listed in Additional file 1: Table S1 [28–30]. *Influenza A virus* and HCoV were the respiratory viruses most frequently detected in the MWPyV-positive patients, which is consistent with their frequent detection with WUPyV and KIPyV in respiratory samples [31]. The detection rate of *Influenza A virus* was 69.2%, followed by HCo-OC43, and then PIV3, the predominant subtype among PIVs. A comparison of the clinical symptoms experienced by the patients with and without co-infections indicated that co-infection with MWPyV did not affect the severity of the illness. 11 MWPyV-positive children with ARIs were diagnosed with bronchopneumonia, and their commonest symptoms were fever and cough. The symptoms of the three MWPyV-positive children with diarrhea who were co-infected with *Rotavirus* included diarrhea and fever. Our data suggest that primary MWPyV infections occur in childhood, predominantly sporadically. However, it is possible that MWPyV is shed within both the gastrointestinal and respiratory tracts. Understanding these factors will help us prevent the transmission of MWPyV infections. To our knowledge, this is the first report of the epidemiological and clinical profiles of MWPyV in children hospitalized with ARIs or diarrhea in China, based on a real-time qPCR specific for MWPyV.

## Conclusions

Real-time PCR is an effective tool for the detection of MWPyV in different types of samples. MWPyV infection mainly occurs in young children, and fecal–oral transmission is a possible route of its transmission.

## Additional file

Additional file 1:
**Table S1.** Primers and probes used to detect respiratory viruses. **Table S2.** Primers used to amplify the VP1 complete genome with nested PCR. (DOCX 16 kb)

## Abbreviations

ARIs: acute respiratory tract infections
HPyVs: Human polyomaviruses
MWPyV: Human Malawi polyomavirus
NPA: nasopharyngeal aspirate
qPCR: quantitative PCR
