# Supplementary material for: Quantitative detection of human Malawi polyomavirus in nasopharyngeal aspirates, sera, and feces in Beijing, China, using real-time TaqMan-based PCR
Source: Virol J. 2017 Aug 14;14:152. doi: 10.1186/s12985-017-0817-2 (PMC5557062; doi:10.1186/s12985-017-0817-2)
Supplement: Additional file 1: — Table S1. Primers and probes used to detect respiratory viruses. Table S2. Primers used to amplify the VP1 complete genome with nested PCR. (DOCX 16 kb) [file 12985_2017_817_MOESM1_ESM.docx]

**Table S1 Primers and probes used to detect respiratory viruses**

| Assay | Primer and Probe (5′-3′) | Reference | |
| --- | --- | --- | --- |
| RSV-Forward | GGCAMTATGGAAACATACGTGAA | | 29 |
| RSV-Reverse | TCTTTTTCTAGGACATTGTAYTGAACAG | |  |
| RSV-Probe | FAM-CTGTGTATGTGGAGCCTTCGTGAAGCT-BHQ1 | |  |
| IFVA-Forward | GACCRATCCTGTCACCTCTGAC | | 29 |
| IFVA-Reverse | AGGGCATTYTGGACAAAKCGTCTA | |  |
| IFVA-Probe | FAM-TGCAGTCCTCGCTCACTGGGCACG-BHQ1 | |  |
| IFVB-Forward | GAGACACAATTGCCTACCTGCTT | | 30 |
| IFVB-Reverse | TTCTTTCCCACCGAACCAAC | |  |
| IFVB-Probe | FAM-AGAAGATGGAGAAGGCAAAGCAGAACTAGC-TAMRA | |  |
| PIV1-Forward | AGTTGTCAATGTCTTAATTCGTATCAAT | | 29 |
| PIV1-Reverse | TCGGCACCTMGTMTTTTGAGTT | |  |
| PIV1-Probe | FAM-ATAGGCCAMGATTGTTGTCGAGACTATTCCAA-BHQ1 | |  |
| PIV2-Forward | GCATTTCCAATCTACAGGACTATGA | |  |
| PIV2-Reverse | ACCTCCTGGTATAGCAGTGACTGAAC | |  |
| PIV2-Probe | FAM-CCATTTMCTMGTGATGGAATCMTCGCMA-BHQ1 | |  |
| PIV3-Forward | TGGYTCAATCTCAACAACAAGATTTAAG | |  |
| PIV3-Reverse | TACCCGAGAAATATTATTTTGCC | |  |
| PIV3-Probe | FAM-CCCRTCTG"T"TGGACCAGGGATATACTACAAA-BHQ1 | |  |
| PIV4-Forward | CTGCCAAATCGGCAATTAAAC | |  |
| PIV4-Reverse | CTGGCAGCAATCATAAGRTGATTC | |  |
| PIV4-Probe | FAM-CATTATTATCTCTGCtTTCCTTACAGGCCACATCA-BHQ1 | |  |
| HBoV-Forward | GCAAATCTCTTCTGGCTACACG | | 31 |
| HBoV-Reverse | CCTCTGCGATCTCTATATTGAAGG | |  |
| HBoV-Probe | FAM-ATGTTGCCGCCAGTAACTCCACCC-TAMRA | |  |
| HMPV-Forward | CATATAAGCATGCTATATTAAAAGAGTCTC | | 29 |
| HMPV-Reverse | CCTATTTCTGCAGCATATTTGTAATCAG | |  |
| HMPV-Probe | FAM-TGYAATGATGAGGGTGTCACTGCGGTTG-TAMRA | |  |
| NL63-Forward | AGGACCTTAAATTCAGACAACGTTCT | | 30 |
| NL63-Reverse | GATTACGTTTGCGATTACCAAGACT | |  |
| NL63-Probe | FAM-TAACAGTTTTAGCACCTTCCTTAGCAACCCAAACA-TAMRA | |  |
| OC43-Forward | GCTCAGGAAGGTCTGCTCC | |  |
| OC43-Reverse | TCCTGCACTAGAGGCTCTGC | |  |
| OC43-Probe | FAM –TTCCAGATCTACTTCGCGCACATCC-TAMRA | |  |
| HKU1-Forward | AGTTCCCATTGCTTTCGGAGTA | |  |
| HKU1-Reverse | CCGGCTGTGTCTATACCAATATCC | |  |
| HKU1-Probe | FAM -CCCCTTCTGAAGCAA-MGB | |  |
| 229E-Forward | CGCAAGAATTCAGAACCAGAG | |  |
| 229E-Reverse | GGCAGTCAGGTTCTTCAACAA | |  |
| 229E-Probe | FAM–CCACACTTCAATCAAAAGCTCCCAAATG-TAMRA | |  |

**Table S2** **Primers used to amplify the VP1 complete genome with nested PCR**

| Number | Primer sequences (5′-3′) | | Amplicon size (bp) |
| --- | --- | --- | --- |
| 1 | | TGTTCCAGATCTTGCAGAATGTC | 363 |
|  |  | GCTAGTAACTCCTATTAATTCAG |  |
|  |  | TCCAGGAGGAGCAAGACAAAGAC |  |
|  |  | GCTTTAAATGAGGCATCATAGGTT |  |
| 2 | | AACTGAATTAATAGGAGTTACTAGC | 333 |
|  |  | CTCCAGTAACAGTTCCATAGTATCT |  |
|  |  | CAGCTGCCCACTTATAGTACAGCTA |  |
|  |  | TCCACAAATATCTACTGCTGTTACA |  |
| 3 | | AGATACTATGGAACTGTTACTGGAG | 509 |
|  |  | CAAGACTCTGGAATTAATTCACAAT |  |
|  |  | TTGCAGTGGGAGGACAGCCTTTAGA |  |
|  |  | TGCTTCTTTGTTGGTGGCAACCTGT |  |
| 4 | | AGGAGGAGCAAGACAAAGACAGA | 226 |
|  |  | GACATTCTGCAAGATCTGGAACA |  |
|  |  | CCTTACTCCTCCACAAGTAAGGT |  |
|  |  | GGTTGAAGAATGACCTCAACTGTC |  |

Note: The first pair of primer is outer primer; the second is inner primer in every group
